# Effectiveness of digital and mobile-based interventions on sleep quality among nurses: a systematic review and meta-analysis

**DOI:** 10.3389/fdgth.2026.1774094

**Published:** 2026-04-24

**Authors:** Fenglan Lun, Wei Wei, Jinping Dong, Xiaohong Cui, Xueying Ding, Hongxia Yang, Xiaoyan Sun

**Affiliations:** 1 Medical College, Weifang University of Science and Technology, Shandong, Weifang, China; 2 Neurology Department, Weifang People’s Hospital, Shandong, Weifang, China

**Keywords:** digital health, eHealth, meta, nurses, sleep quality

## Abstract

**Background:**

Nurses frequently endure diminished sleep quality, sleeplessness, and psychological distress due to high-intensity shifts and persistent work pressure. Digital health interventions are increasingly utilised to enhance sleep behaviour; however, systematic information about their real benefits on the nursing population remains insufficient.

**Objective:**

To assess the efficacy of digital and mobile interventions on sleep and associated psychological consequences in nurses.

**Methods:**

This review adhered to Cochrane principles and PRISMA standards. A multitude of databases were examined, including PubMed, Web of Science, the Cochrane Library, Embase, Scopus, and EBSCO. Two reviewers conducted study screening and quality assessment independently. The primary outcomes were the Pittsburgh Sleep Quality Index (PSQI), Insomnia Severity Index (ISI), and Epworth Sleepiness Scale (ESS). The statistical analysis was conducted using RevMan 5.4 software. Continuous outcome variables were aggregated using standardised mean differences (SMD), mean differences (MD), and 95% confidence intervals (CI).

**Results:**

Eleven studies comprising 2,321 nurses were included. Digital interventions markedly enhanced sleep quality (PSQI: MD = −2.94, 95% CI −5.22 to −0.66) and reduced insomnia severity (ISI: MD = −3.32, 95% CI −5.19 to −1.45). A significant disparity was also noted in daytime sleepiness (ESS), with reduced scores in the intervention group. The interventions also diminished depression (SMD = −0.46, 95% CI −0.80 to −0.13), anxiety (SMD = −0.29, 95% CI −0.44 to −0.14), and fatigue (SMD = −0.41, 95% CI −0.75 to −0.07), while no significant effect was found for work-related stress.

**Conclusion:**

Digital and mobile-based interventions seem to enhance sleep quality and psychological well-being in nurses. Nonetheless, due to the significant variability and the restricted number of studies, additional high-quality trials are required to validate these findings.

## Introduction

Nurses hold significant frontline care duties within the global healthcare system. Nonetheless, they encounter obstacles, including elevated occupational stress, prolonged shifts, and disturbed circadian cycles (1). These variables render the sleep quality difficulties of nurses especially pronounced. Studies indicate that a considerable percentage of nurses encounter sleep disruptions, characterised by difficulties in initiating sleep, diminished sleep duration, and irregular sleep architecture. The prevalence of night shifts, workload, and psychological stress continues to be the principal factors influencing nurses' sleep (2). Poor sleep quality is not merely a prevalent subjective issue, but is increasingly acknowledged as a long-term health hazard among nurses. Sleep disturbances in nurses are significantly linked to numerous detrimental outcomes. Epidemiological research indicates that prolonged sleep loss correlates with cardiovascular metabolic disorders and heightened symptoms of despair and anxiety (3). Simultaneously, these adverse consequences can impair attention, executive function, and clinical judgement, thereby impacting nursing quality and patient safety (4). Sleep disturbances can diminish job satisfaction and elevate turnover intentions, thus jeopardising the stability of the nursing workforce and posing a possible risk to the sustainable development of the healthcare system.

Digital and mobile interventions have been extensively employed in recent years to enhance sleep quality and have progressively diversified across various groups (5, 6). Such interventions generally employ smartphone applications, wearable devices, and online platforms to facilitate individuals' self-management of sleep through modules including sleep monitoring, sleep hygiene instruction, cognitive-behavioural methods, relaxation training, and real-time feedback (7). In contrast to conventional face-to-face interventions, digital and mobile health tools offer advantages like enhanced accessibility, reduced costs, and the capacity for utilisation during fragmented time, rendering them especially appropriate for nurses with demanding work schedules and time limitations (8). Nonetheless, current clinical trials and systematic reviews predominantly concentrate on typical adults, individuals with insomnia, or the elderly, while insufficient emphasis is placed on the occupational cohort of nurses (9, 10).

Currently, published evaluations demonstrate considerable variability regarding research subjects and types of interventions. The amalgamated analysis of people with diverse professional histories, work settings, and lifestyles may obscure the influence of occupational characteristics on the efficacy of interventions. Certain studies exclusively assess one form of digital intervention or predominantly emphasise subjective sleep metrics, offering limited analysis of multidimensional markers such as sleep architecture and psychological outcomes (11). These limitations lead to an absence of systematic and quantitative research about the efficacy of digital and mobile sleep interventions for nurses, complicating the establishment of a robust foundation for nursing practice and management decisions. This systematic review and meta-analysis seeks to summarise and quantify the impacts of digital and mobile treatments on nurses' sleep quality, while also comparing the efficacy of various intervention types in enhancing sleep-related outcomes. Examine the possible influence of research design and demographic variables on the efficacy of interventions to establish a foundation for formulating evidence-based sleep intervention strategies for nurses.

## Methods

### Literature search

In alignment with the Preferred Reporting Items for Systematic Reviews and Meta-Analyses (PRISMA) recommendations and the Cochrane Handbook for Systematic Reviews of Interventions (12). A thorough literature search was performed across eight English databases: PubMed, EMBASE, Web of Science, CINAHL, Cochrane Library, Scopus, EBSCO, and Google Scholar (as an auxiliary source). The search encompassed all publications from the creation of the database to May 2025. A mixture of subject headers and free-text phrases was employed to guarantee comprehensive coverage of pertinent studies. The search strategy employed in PubMed was as follows: (nurse OR “nursing staff” OR “clinical nurse”) AND (sleep OR “sleep quality” OR insomnia OR “sleep disorder”) AND (“digital intervention” OR “mobile-based intervention” OR “mobile application” OR “smartphone app” OR “eHealth” OR “mHealth” OR “wearable device” OR “virtual intervention”). All discovered references were imported into EndNote X9 (Clarivate Analytics) for the purpose of duplicate elimination.

Two reviewers (FLL and JPD) conducted independent screenings of titles and abstracts. Subsequently, full-text papers were evaluated based on the established inclusion and exclusion criteria. Disputes arising during the selection process were settled through dialogue or arbitration by a third reviewer (XHC).

### Inclusion and exclusion criteria

Based on the PICO-S framework:

P (Population): Registered nurses or clinical nursing staff aged ≥18 years.
I (Intervention): Digital or mobile interventions, encompassing mobile applications, wearable gadgets, virtual reality, digital relaxation training, and electronic sleep monitoring.
C (Comparison): Usual care, no intervention, or placebo intervention.
O (Outcome): At least one quantitative measure related to sleep quality, such as the Pittsburgh Sleep Quality Index (PSQI), Insomnia Severity Index (ISI), or Epworth Sleepiness Scale (ESS).
S (Study Design): Studies published in English or Chinese, encompassing RCTs and quasi-experimental designs, without limitations on publication year.

The exclusion criteria were delineated as follows: (1) Studies presented solely as abstracts, duplicate papers, or those without comprehensive data. (2) Articles not published in English or Chinese. Studies were evaluated as possessing inadequate methodological quality.

### Data extraction

Two reviewers independently evaluated the titles and abstracts of all retrieved studies for preliminary selection and eliminated duplicates. The whole texts were subsequently examined for secondary screening to eliminate papers that failed to satisfy the inclusion criteria. Disagreements or uncertainties were addressed through discussion or arbitration by a third reviewer. The extracted data encompassed the first author's name, publication year, geographical location, study design (randomised controlled trial or quasi-experimental study), baseline characteristics of nurse participants (age, gender, years of experience, and department type), sample size and group allocation, specifics of the intervention (mobile applications, virtual reality programs, wearable devices, or online courses), frequency and duration of the intervention, control conditions (usual care, no intervention, or placebo), primary outcome measures (PSQI, ISI, ESS), assessment time points (pre- and post-intervention or follow-up), and principal study findings with effect sizes. To ensure data accuracy and the methodological rigour of the meta-analysis, efforts will be taken to contact the original study authors via email to acquire supplemental materials when critical information is missing or ambiguous. Two researchers (WW and XYS) independently evaluated the risk of bias utilising the Cochrane Risk of Bias tool, which examines selection bias (random sequence generation and allocation concealment), performance bias, detection bias, attrition bias, reporting bias, and other biases. Designated as Grade A when all domains were assessed to have a low risk of bias, and as Grade B when certain domains were evaluated as low risk. Discrepancies among reviewers were reconciled through conversation with a third reviewer.

### Data analysis

Statistical analyses were performed with RevMan version 5.4. Continuous outcome variables were aggregated using standardised mean differences (SMD), mean differences (MD), and 95% confidence intervals (CI). Model selection was determined by the degree of statistical heterogeneity. If *I*^2^ ≤ 50% and *P* > 0.10, heterogeneity is considered acceptable and a fixed effects model is used. Alternatively, implement a random effects model. Subgroup analyses were performed based on intervention type (application or online course), intervention duration (less than five weeks or five weeks or more), and evaluation instruments to investigate heterogeneity among subgroups. Egger's test for publication bias was performed with Stata 17.1 software. Sensitivity analysis was conducted by re-executing the meta-analysis subsequent to the exclusion of studies exhibiting a high risk of bias. Statistical significance was defined as *P* < 0.05.

## Results

### Search results

**Figure 1:**
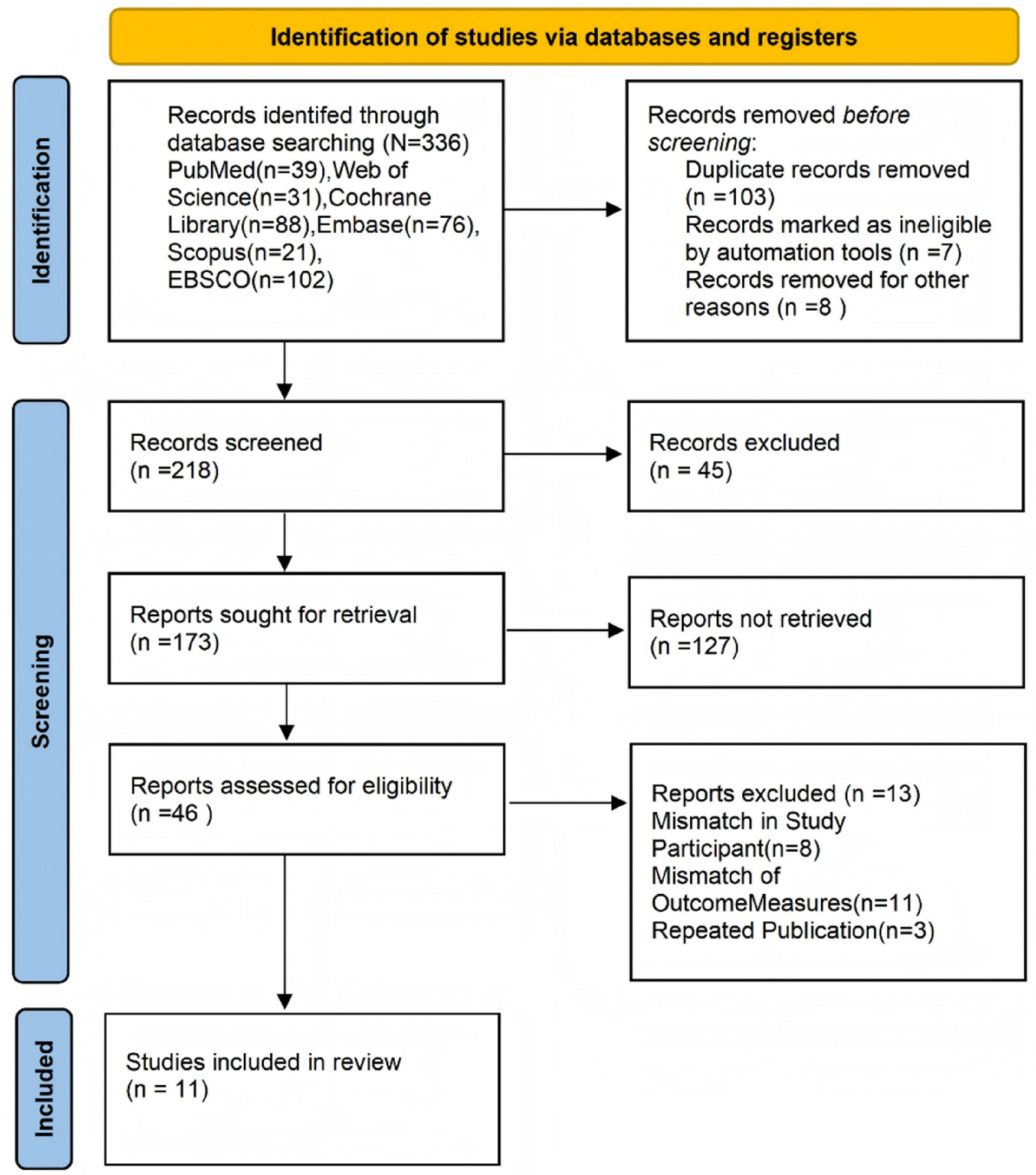
Flow chart of the study selection process.

The search methodology is primarily illustrated in Supplementary Data Sheet 1. Figure 1 illustrates the study selection diagram for the meta-analysis. A total of 336 records were first identified by electronic database searches, comprising PubMed (*n* = 39), Web of Science (*n* = 31), Cochrane Library (*n* = 88), Embase (*n* = 76), Scopus (*n* = 21), and EBSCO (*n* = 102). Following the elimination of 103 duplicate records, 7 ineligible records detected by automated techniques, and 8 further records eliminated for various reasons, 218 records were evaluated based on titles and abstracts. Forty-five records were eliminated due to irrelevance. Subsequently, 173 full-text reports were requested for retrieval; however, 127 could not be acquired due to inadequate data or inaccessible sources. A total of 46 full-text papers were evaluated for eligibility, of which 13 were excluded: 8 due to participant mismatch (non-nurse populations), 11 owing to result inconsistency (absence of sleep-related outcomes), and 3 for duplicate publishing. In conclusion, 11 papers satisfied the inclusion criteria and were incorporated into the final meta-analysis.

### Study characteristics

Table 1 shows the features of the eligible studies. There were 11 research studies published between 2021 and 2025, comprising 10 randomised controlled trials (RCTs) and one quasi-experimental study, involving 2321 nurses from China, Germany, South Korea, Türkiye, Singapore, Australia, and other countries. The participants are primarily registered nurses and shift nurses.

**Table 1:** Characteristics of included studies.

Study	Country	Design	Sample Size	Intervention Type	Duration	Outcomes
Baek et al. (20)	South Korea	Quasi- experimental	54	Mobile exercise program (aerobic + strength training)	18 weeks	ISI, FFS
Bruckner et al. (13)	Malaysia	RCT	74	Digital CBT-I (6-module program)	6 modules	ISI, ESS, Depression, Work Stress
Celik et al. (18)	Türkiye	RCT	101	Online laughter yoga intervention	4 weeks	PSQI
Ell et al. (14)	Germany	RCT	46	Digital CBT-I with personalized feedback	8 weeks	ISI, ESS, Depression, Anxiety, Work Stress
Fiol-DeRoque et al. (21)	Spain	RCT	482	Mobile psychoeducation + mindfulness intervention	2 weeks	ISI, Depression, Anxiety, Work Stress
Guo et al. (22)	China	RCT	134	Internet-delivered CBT-I/MBSR	8 weeks	ISI, Depression
Ha et al. (16)	South Korea	RCT	60	Mobile wellness program (activity tracking + coaching)	12 weeks	PSQI, FFS
Keng et al. (15)	Malaysia	RCT	80	Mindfulness app-based intervention	3 weeks	PSQI, Depression, Anxiety, FFS
Lu et al. (17)	China	RCT	145	ACT-based digital program	5 weeks	PSQI, Depression, Anxiety
Shriane et al. (23)	Australia	RCT	58	Sleep health mobile application (10 modules)	2 weeks	ISI, ESS, FFS
Wang et al. (19)	China	RCT	401	Internet-based ACT (iACT, 6 lessons)	4 weeks	PSQI

CBT-I, cognitive behavioral therapy for insomnia; ACT, acceptance and commitment therapy; MBSR, mindfulness-based stress reduction; PSQI, Pittsburgh Sleep Quality Index; ISI, Insomnia Severity Index; ESS, Epworth Sleepiness Scale; CES-D-15, 15-item Center for Epidemiological Studies Depression Scale; BDI-II, Beck Depression Inventory-II; DASS-21, Depression, Anxiety, and Stress Scale-21; PHQ-9, Patient Health Questionnaire-9; GAD-7, Generalized Anxiety Disorder 7-item Scale; STAI, State–Trait Anxiety Inventory; FSS, Fatigue Severity Scale; PSS-10, Perceived Stress Scale-10.

To improve conceptual clarity, digital interventions were further classified according to their primary mechanisms: (1) cognitive behavioural therapy-based interventions (CBT-I or internet-based CBT), (2) mindfulness-based interventions, (3) Acceptance and Commitment Therapy (ACT)-based interventions, and (4) digital wellness or lifestyle programs focusing on physical activity and health promotion. The duration of intervention spans from two weeks to 18 weeks. Several trials, such as those by Brückner et al. (13) and Ell et al. (14), used personalised feedback mechanisms and dynamic monitoring to improve user engagement and adherence. Control groups throughout studies frequently comprised waiting, usual care, or no-intervention groups, but a few used active comparators, such as digital psychoeducation or cognitive training apps (15).

### Literature quality assessment

**Figure 2:**
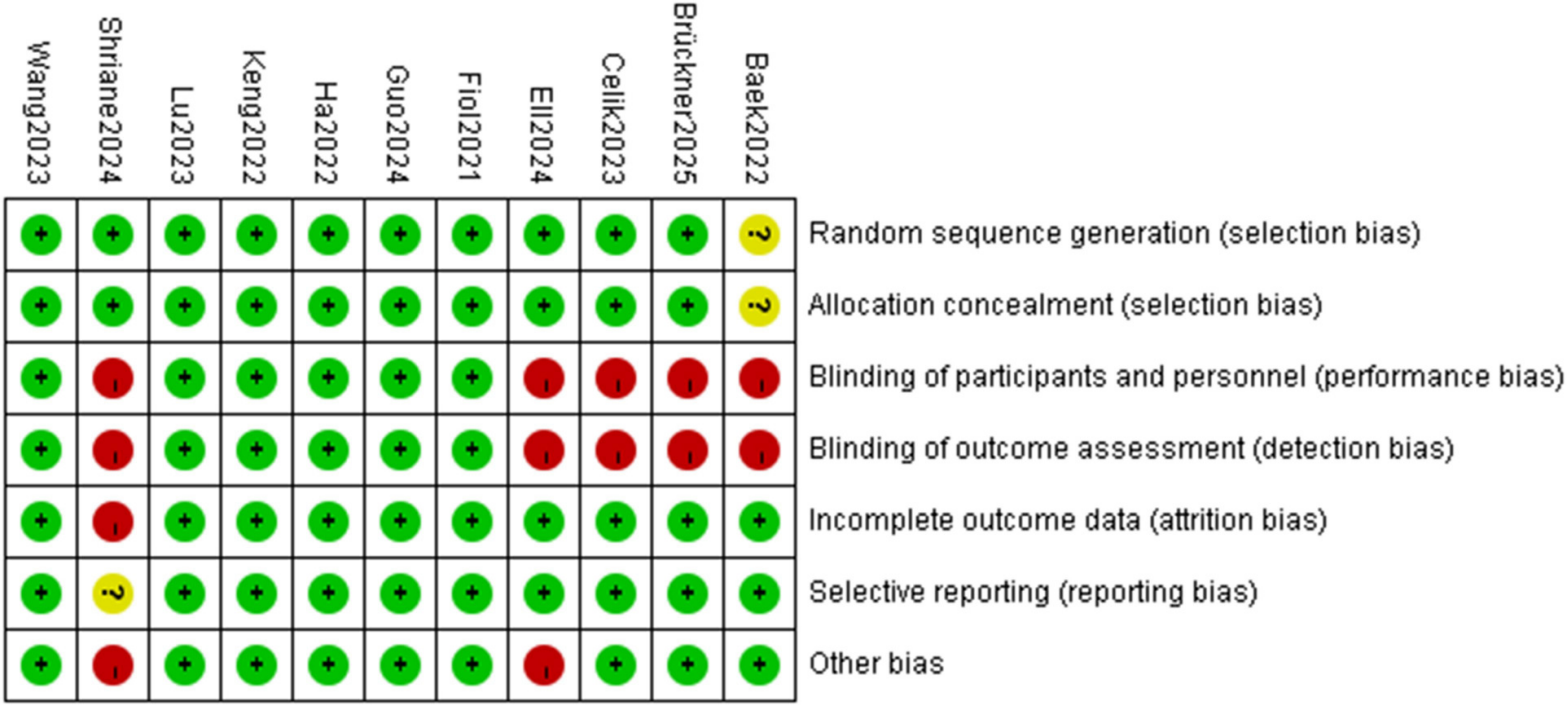
Risk of bias graph.

Figure 2 shows the findings of the risk of bias evaluation. Approximately 73% of the included studies were judged as having a low risk of bias in random sequence generation, with a handful being classified as ambiguous due to poor reporting. Approximately 64% of studies demonstrated low risk in allocation concealment, with a small proportion deemed ambiguous. Because of the sleep interventions, blinding was frequently difficult to apply, resulting in a consistently high risk of performance bias across virtually all investigations, and several studies also revealed a high risk in blinding of outcome assessment (detection bias).

The majority of studies were rated as low risk for incomplete outcome data, selective reporting, and other biases, implying that data were mainly complete and regularly recorded. Overall, 67% of the studies were classed as Grade A (low risk), whereas 33% were classified as Grade B (ambiguous or high risk in specific domains). The overall methodological quality was good, with the main source of bias being limitations in blinding techniques.

### Meta-analysis results

#### PSQI

**Figure 3:**
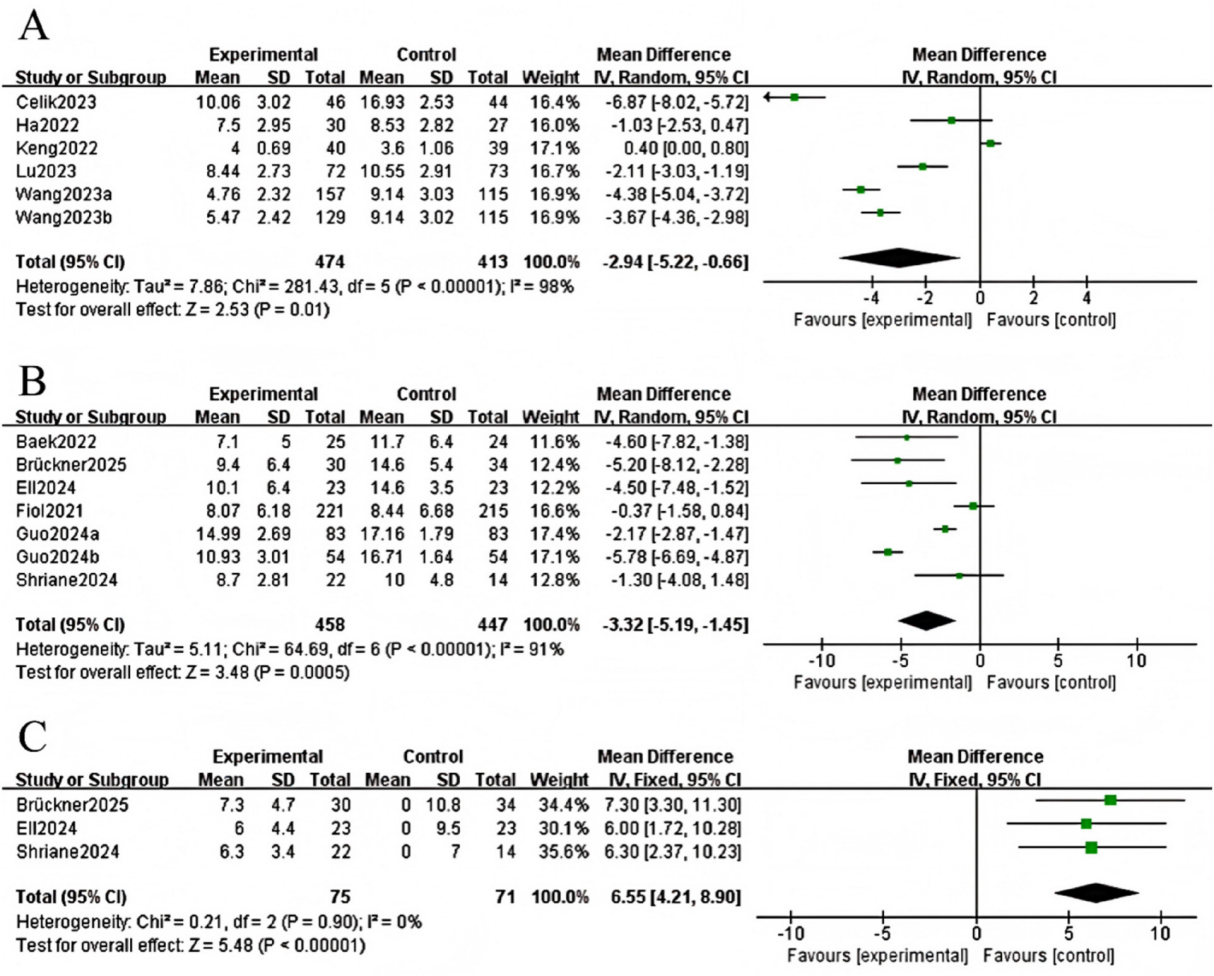
The forest plot shows PSQI,ISI,ESS.

The meta-analysis of the outcome PSQI changes included 5 studies, totalling 474 participants in the experimental group and 413 people in the control group (15–19). High heterogeneity was reported across the trials (*I*^2^ = 98%, *P* < 0.001); therefore, a random-effects model was used. Potential sources of heterogeneity were investigated further using subgroup analyses. The findings indicated that digital and mobile interventions significantly improved sleep quality (MD = −2.94, 95% CI: −5.22 to −0.66, Z = −2.53, *P* = 0.01; Figure 3A).

#### ISI

Six studies reported insomnia outcomes, comprising 458 participants in the experimental and 447 people in the control groups (1, 13, 14, 20–22). Due to substantial heterogeneity among studies (I^2^ = 91%, *P* < 0.0001), so a random-effects model was utilised. The results revealed that digital and mobile interventions had a statistically significant positive effect on insomnia scores compared to the control group (MD = −3.32, 95% CI: −5.19 to −1.45, Z = −3.48, *P* = 0.0005; Figure 3B).

#### ESS

Three studies produced sleepy findings, with a total of 75 participants in the experimental group and 71 people in the control group (13, 14, 23). A fixed-effects model was adopted due to minimal heterogeneity (I^2^ = 0%, *P* = 0.90). The pooled information revealed a statistically significant difference between the groups (MD = 6.55, 95% CI 4.21 to 8.90, *P* < 0.00001, Figure 3C). Higher ESS scores imply increased daytime sleepiness. This finding shows that the intervention group had lower ESS ratings, indicating less daytime sleepiness.

### Depression

**Figure 4:**
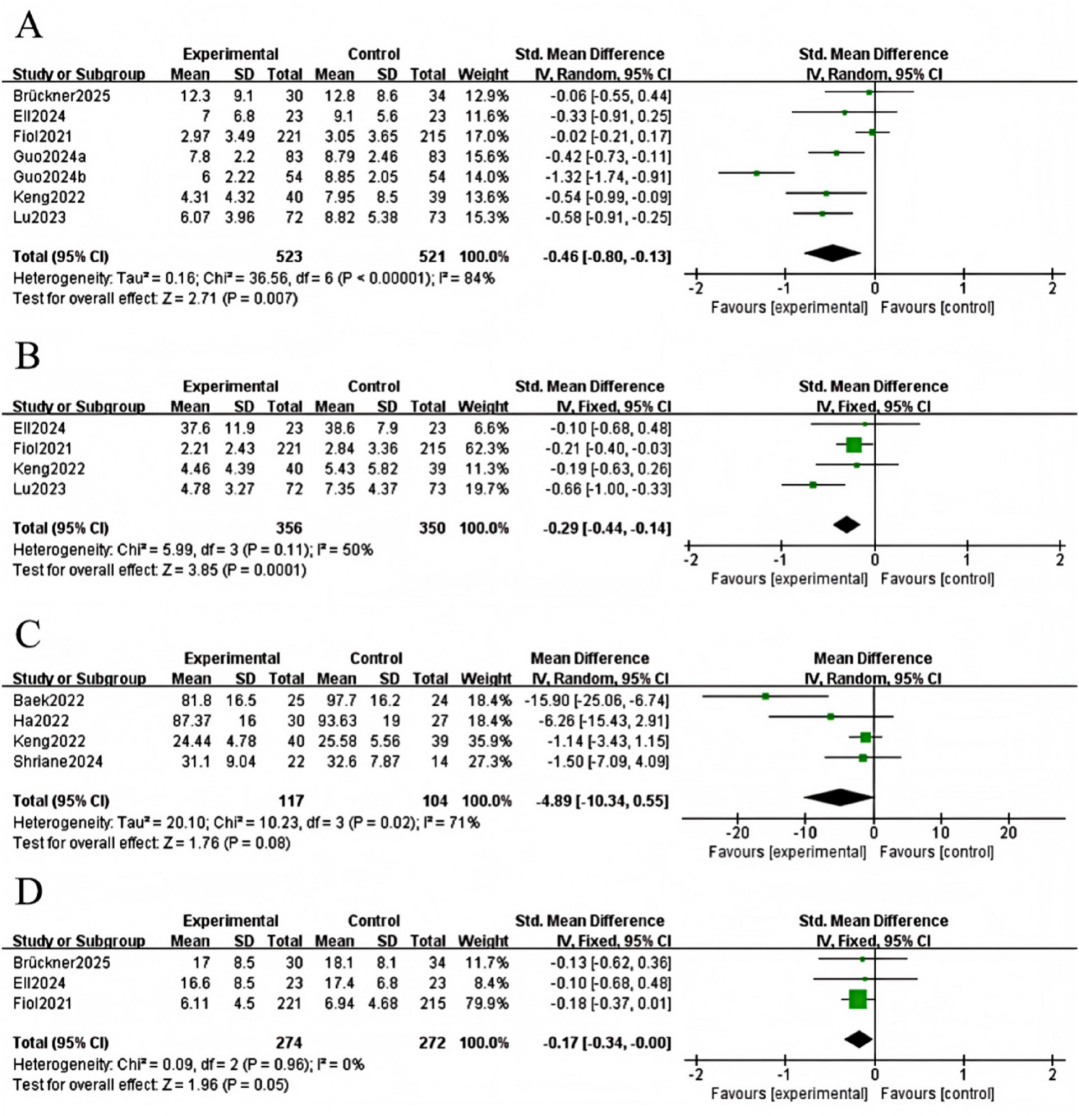
The forest plot shows depression, anxiety, fatigue, and work stress.

Six studies examined depression levels, comprising 523 participants in the experimental group and 521 people in the control group (13–15, 17, 21, 22). Heterogeneity among studies was high (I^2^ = 84%, *P* < 0.0001), so a random-effects model was used. Results demonstrated that digital and mobile interventions led to a significant reduction in depressive symptoms when compared to the control group (SMD=−0.46, 95% CI: −0.80 to −0.13, Z = −2.71, *P* = 0.007; Figure 4A).

### Anxiety

Four studies examined anxiety levels, involving 356 participants in the experimental group and 350 in the control group (14, 15, 17, 21). Heterogeneity among studies was moderate (I^2^ = 50%, *P* = 0.11), so a fixed-effects model was used. The results demonstrated that digital and mobile interventions significantly improved anxiety symptoms compared to the control group (SMD=−0.29, 95% CI: −0.44 to −0.14, Z = −3.85, *P* = 0.0001; Figure 4B).

### Fatigue

Four studies examined fatigue levels, involving 104 participants in the control group and 117 in the experimental group (15, 16, 20, 23). Heterogeneity among studies was low (I^2^ = 33%, *P* = 0.21), so a fixed-effects model was used. Results demonstrated that digital and mobile interventions led to a significant reduction in fatigue when compared with the control group (SMD=−0.41, 95% CI: −0.75 to −0.07, Z = −2.40, *P* = 0.02; Figure 4C).

### Work stress

Three studies examined the effects of occupational stress, involving 274 participants in the experimental group and 272 in the control group (13, 14, 21). Due to the low heterogeneity observed between the studies (*I*^2^ = 0%, *P* = 0.96), a fixed-effects model was used for the analysis. The results revealed that digital and mobile interventions did not significantly lower work stress compared to the control group (SMD = −0.17, 95% CI: −0.34 to 0.00, Z = −1.96, *P* = 0.05; Figure 4D).

### Assessment of publication bias

**Figure 5:**
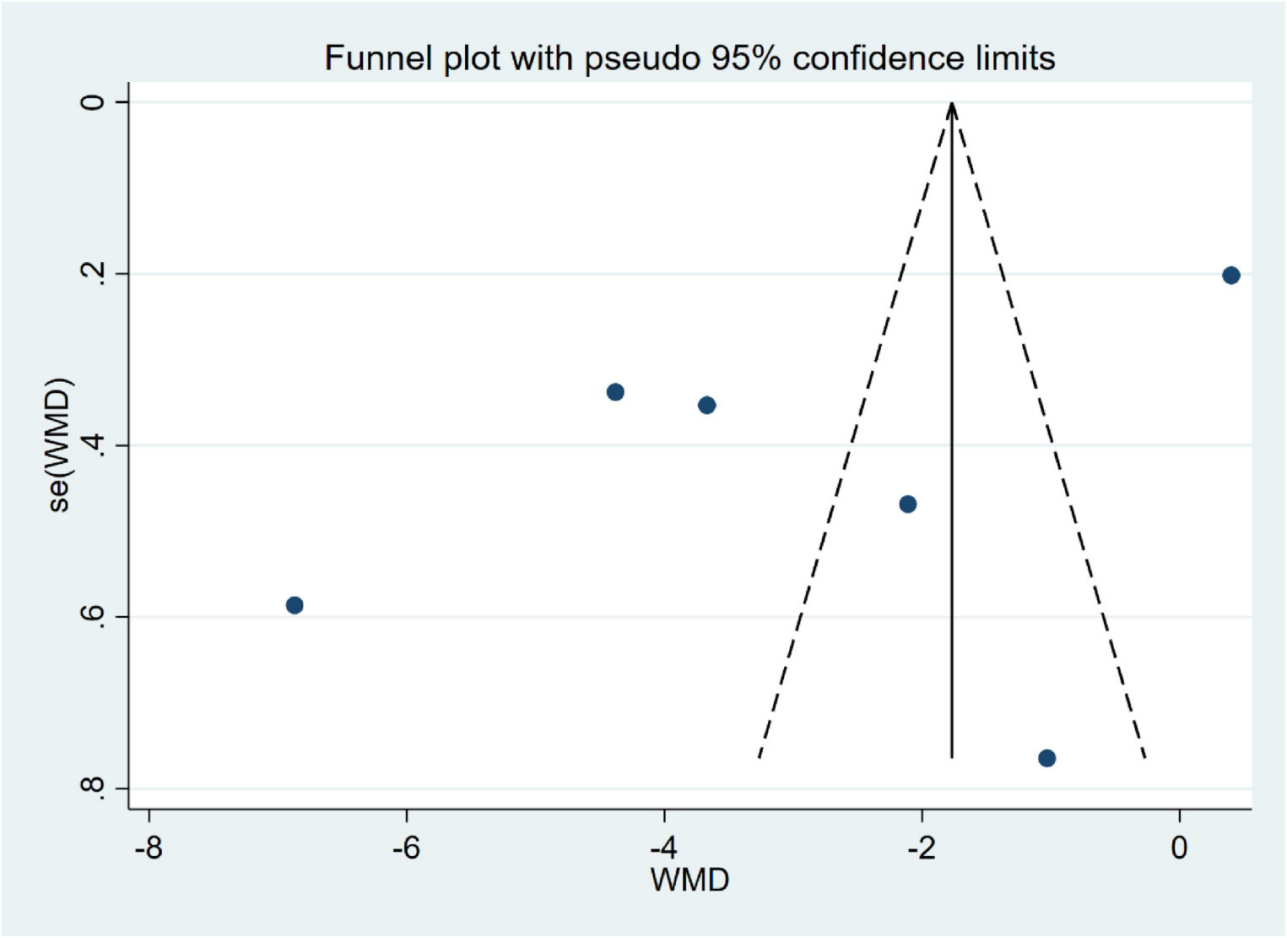
Funnel plot of PSQI.

The Egger's test for sleep quality, insomnia, and depression (*P* > 0.05) revealed no substantial publication bias. The visual asymmetry of the sleep quality funnel plot indicates probable publication bias or small sample research effects on this outcome (Figure 5). 

### Sensitivity analyses

Sensitivity analyses were carried out progressively, removing studies having a high or unclear risk of bias and re-running the meta-analysis. The overall direction and statistical significance of the pooled estimates remained virtually constant, indicating that the results were fairly resilient.

### Subgroup analysis

#### PSQI

**Figure 6:**
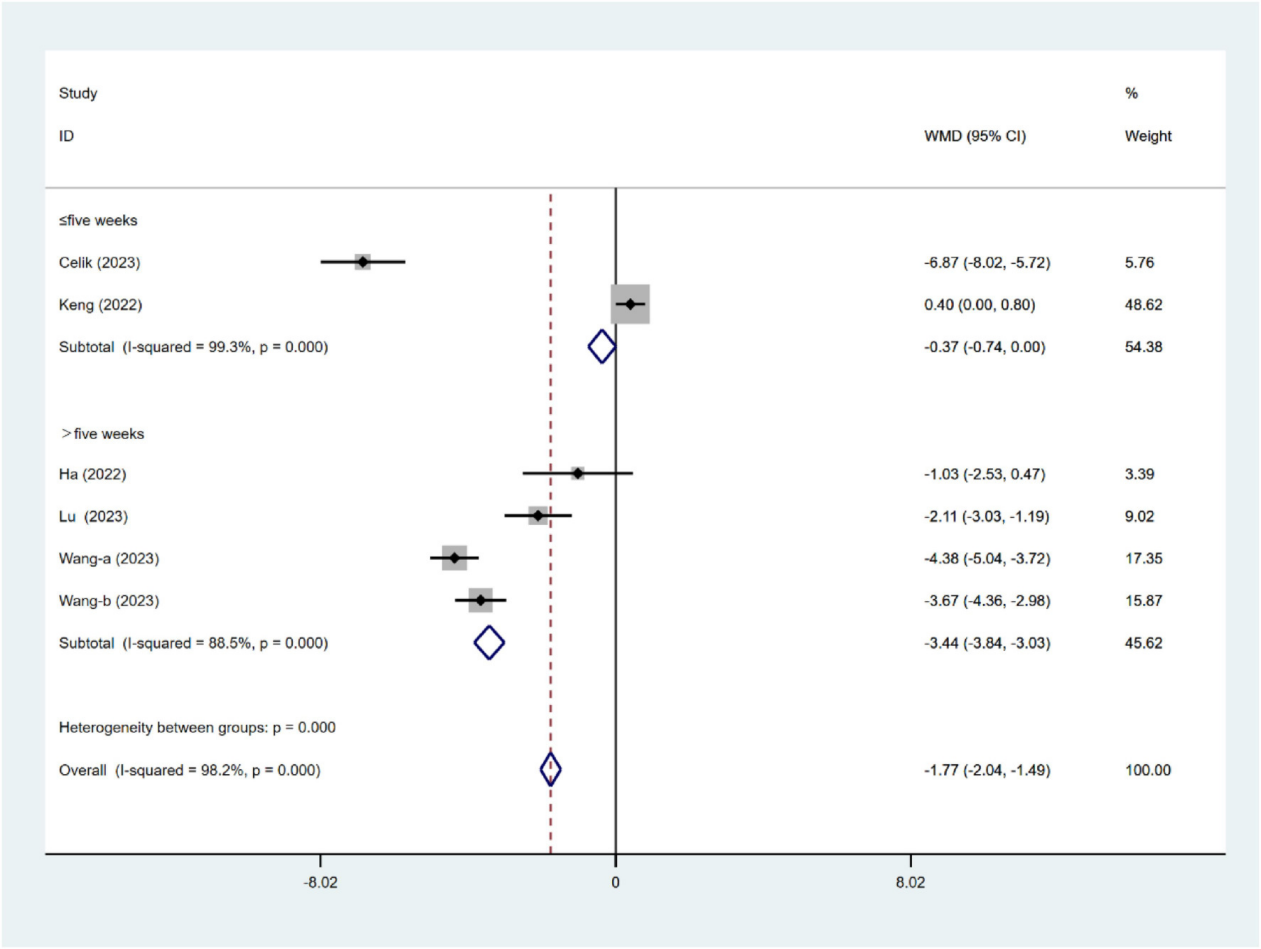
Subgroup analysis of PSQI intervention duration.

As illustrated in Figure 6, subgroup analysis revealed a statistically significant difference in sleep quality outcomes for treatments of different durations (*P* < 0.001). Compared with interventions lasting ≤5 weeks (WMD = −0.37, 95% CI: −0.74 to 0.00), those lasting >5 weeks were significantly associated with improvements in nurse sleep quality (WMD=−3.44, 95% CI: −3.84 to −3.03). Despite the between-group difference, significant heterogeneity persisted within both categories.

**Figure 7:**
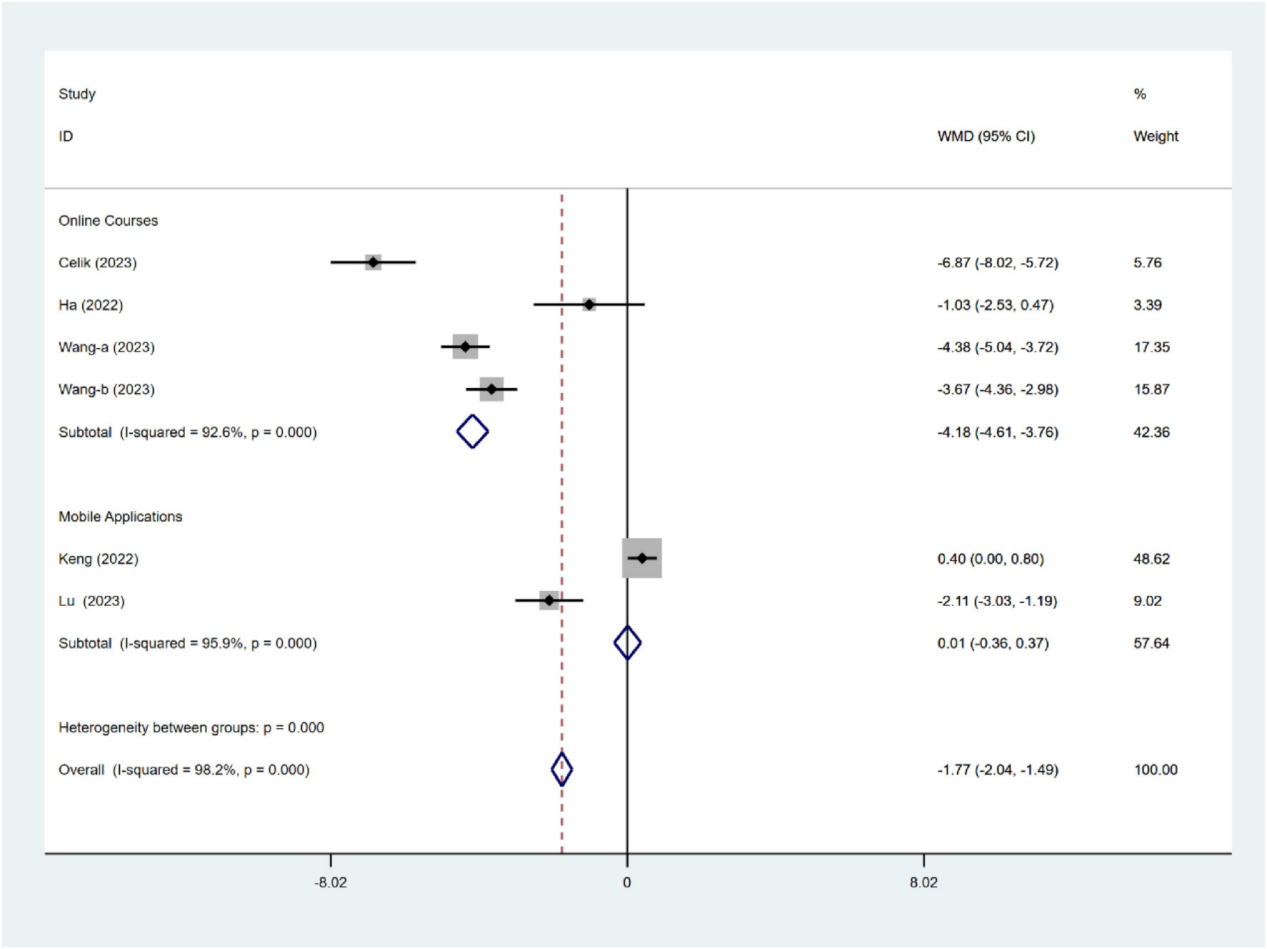
Subgroup analysis of PSQI intervention types.

Similarly, subgroup comparisons based on intervention type revealed a significant difference (*P* < 0.001; Figure 7). Intervention measures in online courses (WMD = −4.18, 95% confidence interval: −4.61 to −3.76) are more effective in enhancing sleep quality than those in mobile applications. (WMD = 0.01, 95% CI: −0.36 to 0.37). However, significant heterogeneity was found within both subgroups.

#### ISI

**Figure 8:**
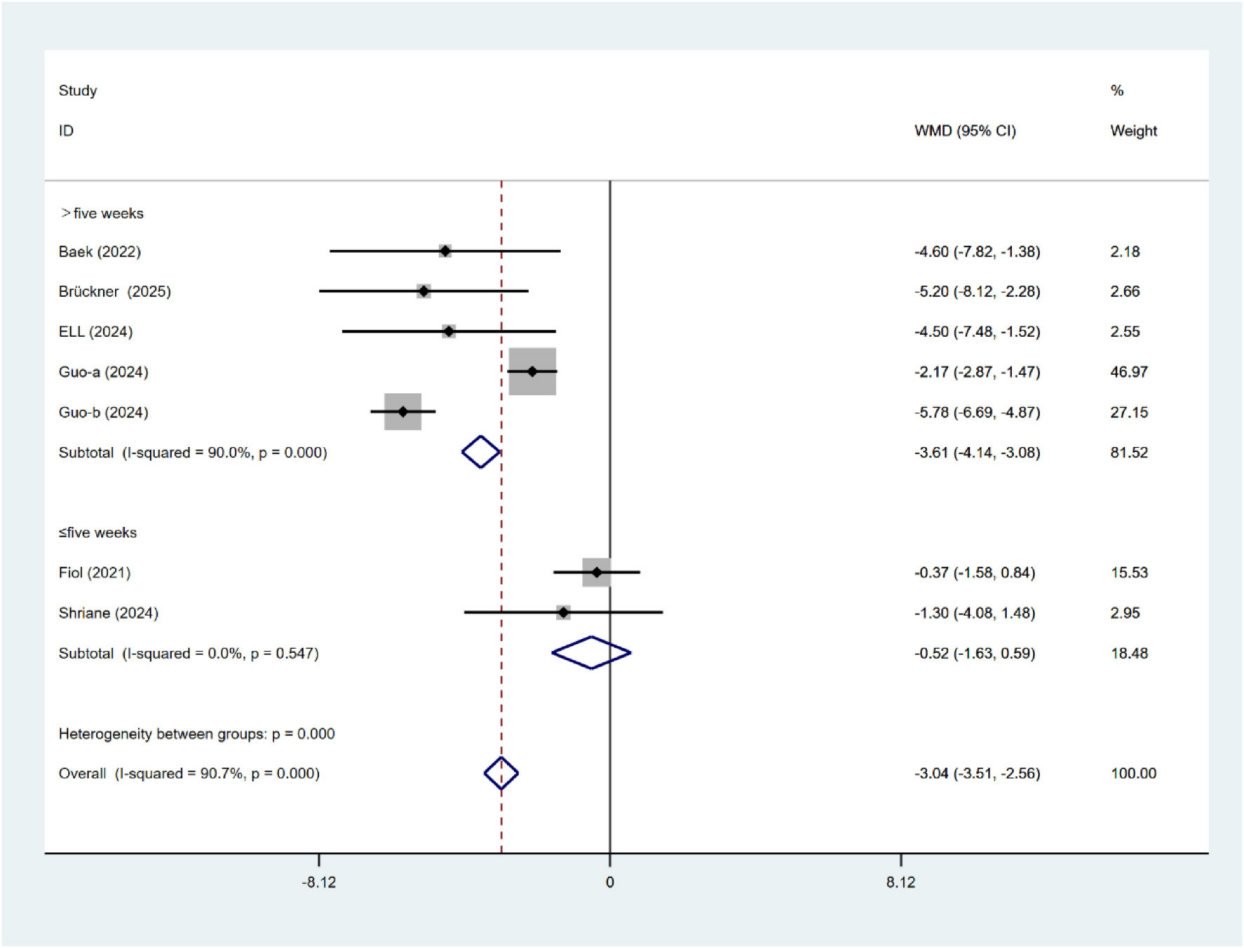
Subgroup analysis of ISI intervention duration.

**Figure 9:**
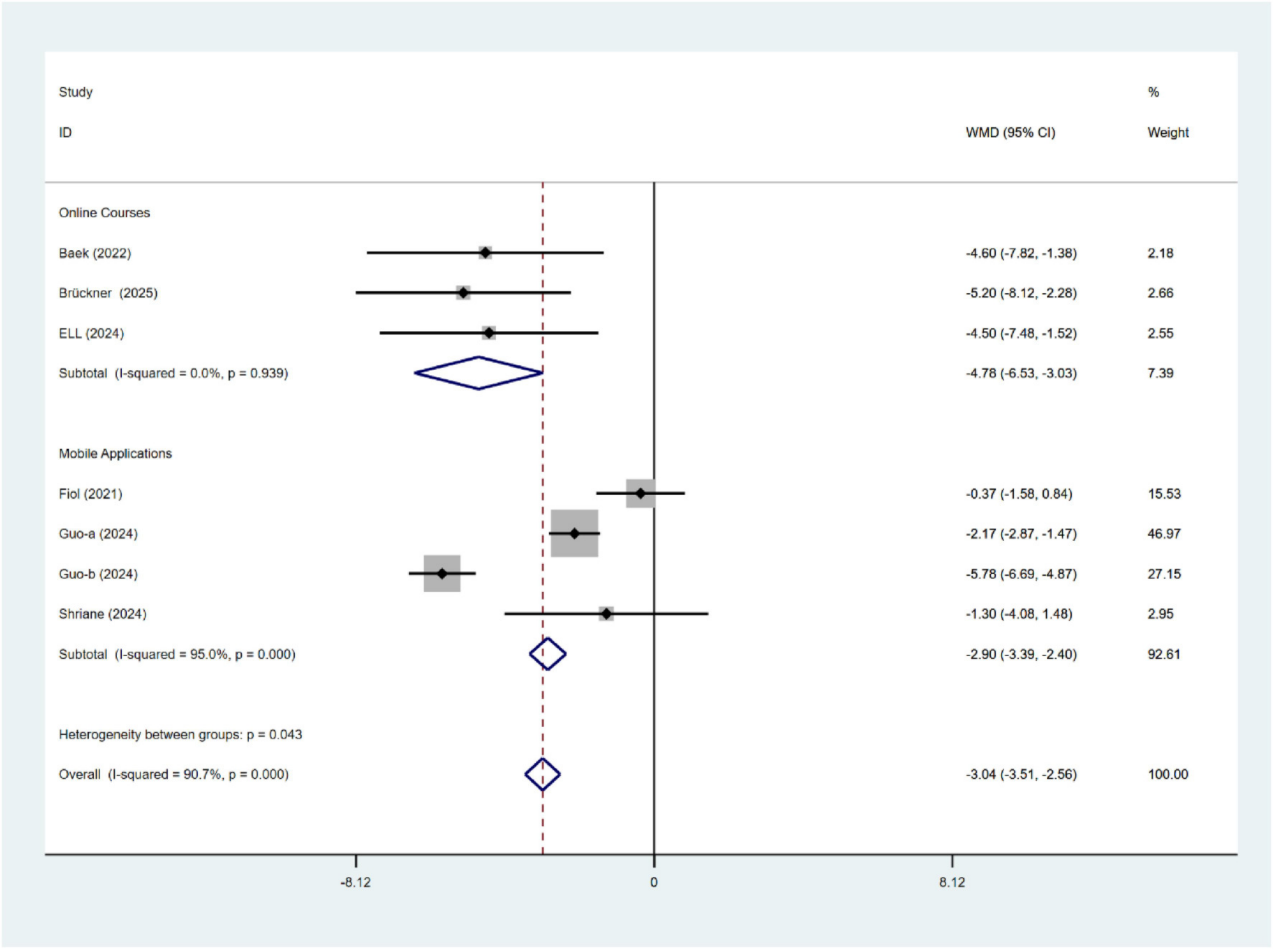
Subgroup analysis of ISI intervention types.

As indicated in Figure 8, the duration of the intervention had a substantial effect on the alleviation of insomnia symptoms (*P* < 0.001). Interventions lasting >5 weeks demonstrated superior efficacy (WMD = −3.61, 95% CI: −4.14 to −3.08) compared with those ≤5 weeks (WMD = −0.52, 95% CI: −1.63 to 0.59). The duration of the two procedures varies significantly. As indicated in Figure 9, the intervention type has a substantial effect on the insomnia status of nurses (*P* = 0.043). Compared with mobile applications, online courses (WMD = −4.78, 95% CI: −6.53 to −3.03) significantly reduced insomnia symptoms (WMD = 2.90, 95% CI: −3.39 to −2.40). There is significant heterogeneity between subgroups.

### Depressive

**Figure 10:**
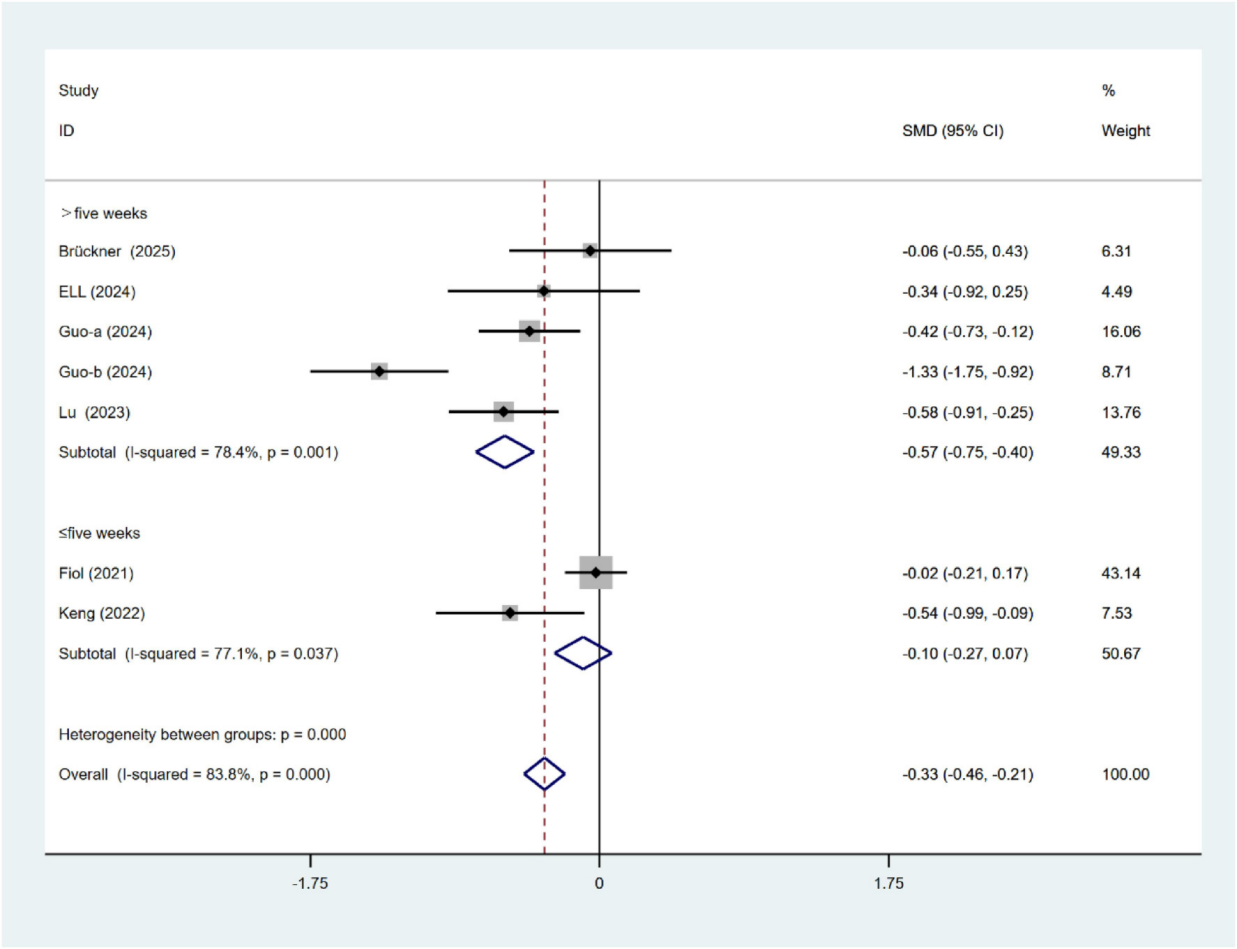
Subgroup analysis of depressive intervention duration.

**Figure 11:**
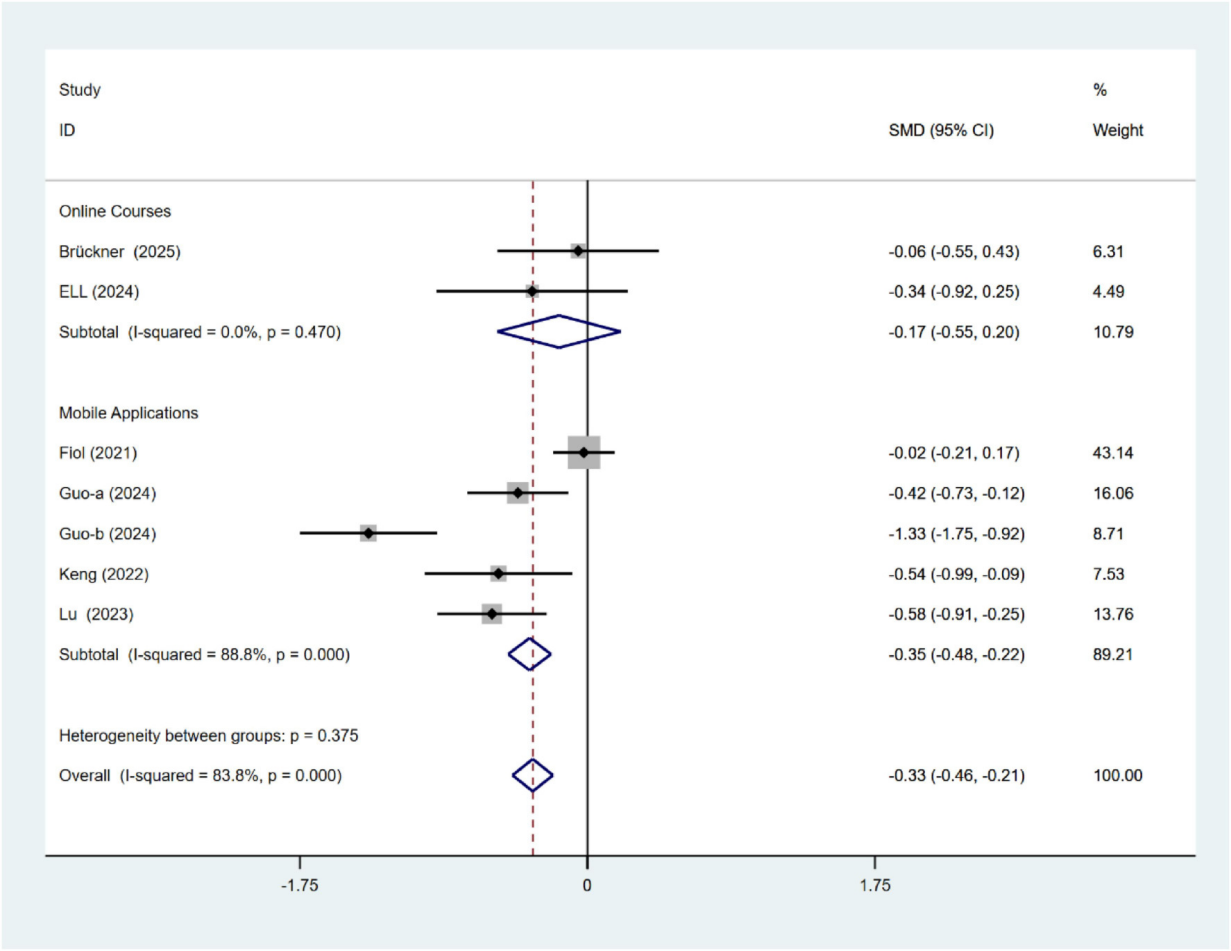
Subgroup analysis of depressive intervention types.

For depressive symptoms, significant subgroup differences were observed based on the duration of intervention (*P* < 0.001; Figure 10). Interventions lasting for more than 5 weeks can significantly alleviate depressive symptoms (SMD = −0.57, 95% CI: 0.75 to −0.40). There were no statistically significant differences between the intervention types (*P* = 0.375; Figure 11). Mobile application intervention (SMD = −0.35, 95% CI: 0.48 to −0.22) demonstrated a substantial reduction in depressive symptoms, whereas the effect of online course-based intervention (SMD = −0.17, 95% CI: −0.55 to 0.20) did not approach statistical significance.

## Discussion

This systematic review and meta-analysis assessed the efficacy of digital and mobile-based interventions for increasing sleep quality and related psychological effects among nurses. The findings show that these therapies significantly improved sleep quality and reduced insomnia severity. There were also benefits in daytime sleepiness, sadness, anxiety, and fatigue. However, no significant effect was observed for work-related stress. These findings indicate that digital interventions may be a realistic and scalable technique for addressing sleep issues among nurses working in demanding clinical contexts.

The current data show that digital interventions can dramatically improve sleep quality in nurses. This conclusion is consistent with earlier studies showing that digital cognitive behavioural therapy and mobile-based sleep programs can successfully enhance sleep outcomes in different groups (24). Similarly, Vallières et al. (25). found that mobile mindfulness interventions significantly reduced cognitive arousal before bedtime, leading to increased subjective sleep pleasure. The results of the subgroup analysis indicate that intervention duration is an important factor in determining intervention effectiveness. Programs lasting more than five weeks produced higher improvements in sleep outcomes than shorter interventions. This finding is consistent with behavioural sleep medicine concepts, which emphasise the need of long-term behavioural change for improved sleep. Behavioural change related to sleep habits usually takes time to consolidate (26). Short-term interventions can raise awareness, but they may not provide people the time to build and maintain stable behavioural changes. The study also implies that organised online course-based interventions may have greater impact than mobile application-based interventions. One probable explanation is that organised prigrammes frequently provide guided modules, personalised feedback, and more systematic learning paths. These components may improve participant engagement and adherence, which are crucial in behavioural interventions (27). In contrast, mobile applications may rely more heavily on user incentive and so have lesser adherence over time (28). Structured digital interventions, such as reminders, feedback, and guided modules, may be more helpful in encouraging long-term behavioural change for nurses with demanding schedules.

Regarding psychological outcomes, the current study discovered that digital health treatments were associated with moderate improvements in depression and anxiety symptoms among nurses, which is consistent with earlier studies. A randomised controlled trial of critical care unit nurses found that internet-based cognitive behavioural therapy significantly lowered depression and anxiety levels (29). Similarly, digital mindfulness programs have been demonstrated to decrease emotional weariness and negative affect by promoting emotional control and self-awareness (30). Bulaj et al. (31) found that interventions offered via mobile platforms, such as meditation and breathing exercises during night shifts or rest periods, can help restore psychological balance and reduce fatigue. These impacts could be explained by how different digital techniques alter cognitive and emotional processes. Cognitive behavioural interventions focus largely on maladaptive ideas and expectations, reducing negative cognitive bias and emotional reactivity (32, 33). On the other hand, mindfulness-based techniques are expected to lower physiological arousal and rumination, perhaps contributing to anxiety reduction. Together, these systems may improve stress adaptation and psychological resilience in nurses (34).

In addition, this study discovered that digital health interventions were connected with decreased weariness among nurses, indicating a possible function in aiding physical and mental recovery. This outcome is consistent with earlier findings that suggest that digital interventions may indirectly ease fatigue by enhancing sleep efficiency and reducing emotional exhaustion (35. However, no significant effect was detected for work-related stress, which is consistent with previous findings. Occupational stress is determined by many organisational elements, including workload, staffing numbers, and institutional support, that are not easily addressed by individual-level digital treatments (4, 36). Longer intervention durations and workplace-specific tactics are likely to be required for more significant changes (37). Organisational initiatives, such as optimising work schedules and enhancing institutional support, may be more effective in lowering occupational stress among nurses (38).

Despite conducting subgroup analyses, significant heterogeneity persisted across several outcomes. This variability is most likely due to the diversity of the included research. The interventions differed in substance, including cognitive behavioural therapy-based programs, mindfulness, Acceptance and Commitment Therapy (ACT), and broader digital wellness approaches, as well as delivery and implementation, which may have influenced their impact on sleep. Intervention duration also varied significantly, ranging from brief programs lasting a few weeks to extended interventions lasting several months, which could influence the stability and amplitude of behavioural changes. Participant characteristics were similarly diverse, including variations in work environments, shift patterns, workload, and baseline psychological health, all of which are directly related to sleep outcomes and may influence response to interventions. Furthermore, most research relied on self-reported measures such as the PSQI and ISI, which can increase variability in comparison to objective sleep examinations.

### Limitations

This study has significant limitations. The number of randomised controlled trials concentrating especially on nurses was low, indicating a vacuum in the literature. Overall, sample sizes were small, and follow-up periods were brief, making it difficult to establish long-term effects. Although Egger's tests revealed no substantial publication bias for primary outcomes, the small number of papers suggests caution in interpretation. The heterogeneity in interventions, participant characteristics, and outcome measures, together with methodological constraints, highlight the need for careful caution when interpreting the pooled data. In addition, some full-text articles could not be retrieved, which may introduce a risk of selection bias.

## Conclusion

Despite these limitations, the study found that digital health treatments can improve sleep quality and related psychological consequences among nurses. Structured online programs lasting more than five weeks were very successful in improving sleep, reducing insomnia severity, and alleviating daytime sleepiness, whereas shorter or lower-compliance mobile interventions provided just minor benefits. Overall, these findings should be interpreted with caution due to the small number of trials and persisting heterogeneity. Future research should prioritise thorough, multicenter trials to evaluate intervention effectiveness and discover which techniques best enhance sleep quality in nursing populations.
