# Supplementary material for: Effectiveness of digital and mobile-based interventions on sleep quality among nurses: a systematic review and meta-analysis
Source: Front Digit Health. 2026 Apr 24;8:1774094. doi: 10.3389/fdgth.2026.1774094 (PMC13154147; doi:10.3389/fdgth.2026.1774094)
Supplement: Supplementary file 1 [file Datasheet1.docx]

Pubmed 39

#1 "nurses"[MeSH Terms]

#2"nursing staff"[MeSH Terms] OR ("nursing"[All Fields] AND "staff"[All Fields]) OR "nursing staff"[All Fields] OR ("nursing staff"[MeSH Terms] OR ("nursing"[All Fields] AND "staff"[All Fields]) OR "nursing staff"[All Fields])

#3 #1 OR #2 217848

#4 "sleep"[MeSH Terms]

#5 "sleep quality"[MeSH Terms] OR ("sleep"[All Fields] AND "quality"[All Fields]) OR "sleep quality"[All Fields] OR ("insomnia s"[All Fields] OR "sleep initiation and maintenance disorders"[MeSH Terms] OR ("sleep"[All Fields] AND "initiation"[All Fields] AND "maintenance"[All Fields] AND "disorders"[All Fields]) OR "sleep initiation and maintenance disorders"[All Fields] OR "insomnia"[All Fields] OR "insomnias"[All Fields]) OR ("sleep wake disorders"[MeSH Terms] OR ("sleep"[All Fields] AND "wake"[All Fields] AND "disorders"[All Fields]) OR "sleep wake disorders"[All Fields] OR ("sleep"[All Fields] AND "disorder"[All Fields]) OR "sleep disorder"[All Fields])

#6  #4 OR #5 252597

#7 digital intervention[MeSH Terms]

#8 ("mobile-based"[All Fields] AND ("intervention s"[All Fields] OR "interventions"[All Fields] OR "interventive"[All Fields] OR "methods"[MeSH Terms] OR "methods"[All Fields] OR "intervention"[All Fields] OR "interventional"[All Fields])) OR ("mobile applications"[MeSH Terms] OR ("mobile"[All Fields] AND "applications"[All Fields]) OR "mobile applications"[All Fields] OR ("smartphone"[All Fields] AND "app"[All Fields]) OR "smartphone app"[All Fields]) OR ("telemedicine"[MeSH Terms] OR "telemedicine"[All Fields] OR "ehealth"[All Fields]) OR ("mhealth s"[All Fields] OR "telemedicine"[MeSH Terms] OR "telemedicine"[All Fields] OR "mhealth"[All Fields]) OR ("wearable electronic devices"[MeSH Terms] OR ("wearable"[All Fields] AND "electronic"[All Fields] AND "devices"[All Fields]) OR "wearable electronic devices"[All Fields] OR ("wearable"[All Fields] AND "device"[All Fields]) OR "wearable device"[All Fields]) OR (("virtual"[All Fields] OR "virtuality"[All Fields] OR "virtualization"[All Fields] OR "virtualized"[All Fields] OR "virtualizing"[All Fields] OR "virtuals"[All Fields]) AND ("intervention s"[All Fields] OR "interventions"[All Fields] OR "interventive"[All Fields] OR "methods"[MeSH Terms] OR "methods"[All Fields] OR "intervention"[All Fields] OR "interventional"[All Fields]))

#9 #7 OR #8 222176

#10 #9 AND #6 AND #3 39

Cochrane:88

#1 17816 Trials matching (nursing staff) OR (nursing staff) OR ( nurse) in Record Title - (Word variations have been searched)

#2 71328 Trials matching (sleep OR "sleep quality" OR insomnia OR "sleep disorder") in Record Title - (Word variations have been searched)

#3 14851 Trials matching ("digital intervention" OR "mobile-based intervention" OR "mobile application" OR "smartphone app" OR "eHealth" OR "mHealth" OR "wearable device" OR "virtual intervention")in Record Title - (Word variations have been searched)

#4 #1 AND #2 AND #3 88

Embase:76

#1 ('nurse'/exp OR nurse OR 'nursing staff'/exp OR 'nursing staff' OR 'clinical nurse')

#2 (sleep:ab,ti OR 'sleep quality':ab,ti OR insomnia:ab,ti OR 'sleep disorder':ab,ti)

#3 ('digital intervention':ab,ti OR 'mobile-based intervention':ab,ti OR 'mobile application':ab,ti OR 'smartphone app':ab,ti OR 'ehealth':ab,ti OR 'mhealth':ab,ti OR 'wearable device':ab,ti OR 'virtual intervention':ab,ti)

#4 #1 AND #2 AND #3 76

CINAHL 49

((nurse OR "nursing staff" OR "clinical nurse")) AND ((sleep OR "sleep quality" OR insomnia OR "sleep disorder")) AND (("digital intervention" OR "mobile-based intervention" OR "mobile application" OR "smartphone app" OR "eHealth" OR "mHealth" OR "wearable device" OR "virtual intervention"))

Web of Science 31

#1 AND #2 AND #3 31

#3TS=((nurse) OR (nursing staff) OR (clinical nurse)2103283

#2ALL=((sleep) OR (sleep quality) OR (sleep disorder) OR( insomnia) ) 252342

#1ALL=((digital intervention) OR (mobile-based intervention) OR (mobile application) OR (smartphone app) OR (eHealth) OR (mHealth) OR (wearable device) OR (virtual intervention)) 22390

Scopus 21

#1 AND #2 AND #3

TITLE-ABS-KEY ( ( ( nurse ) OR ( (nursing staff ) OR (clinical nurse)  ) ) 3272471
TITLE-ABS-KEY ( ( (sleep) OR ( sleep quality) OR ( sleep disorder) OR (insomnia ) ) ) 576

TITLE-ABS-KEY ( ( (digital intervention ) OR ( mobile-based intervention ) OR (mobile application )OR (smartphone app)OR(eHealth) OR (mHealth) OR (wearable device) OR (insomnia ) ) ) 372818

EBSCO 102

S1:[nurse OR nursing staff OR clinical nurse](https://research.ebsco.com/search/results?db=bth,ccm,cul,ddh,ecn,eue,eric,8gh,lgs,mdc,kah,mlf,pif,bwh,ram,30h,nsm&expanders=concept&limiters=FT:Y&q=nurse%20OR%20nursing%20staff%20OR%20clinical%20nurse&searchMode=boolean&sort=relevance&sqId=sq:71dc84f8-fbd6-4c38-b520-3b23970ac5c1&userDirectAction=true) 849,162

S2:[sleep OR sleep quality OR insomnia OR sleep disorder](https://research.ebsco.com/search/results?db=bth,ccm,cul,ddh,ecn,eue,eric,8gh,lgs,mdc,kah,mlf,pif,bwh,ram,30h,nsm&expanders=concept&limiters=FT:Y&q=sleep%20OR%20sleep%20quality%20OR%20insomnia%20OR%20sleep%20disorder&searchMode=boolean&sort=relevance&sqId=sq:c0a7da8d-723e-4d92-9dff-30c634f98cfd&userDirectAction=true) 204,095

S3:[digital intervention OR mobile-based intervention OR mobile application OR smartphone app OR eHealth OR mHealth OR wearable device OR virtual intervention](https://research.ebsco.com/search/results?db=bth,ccm,cul,ddh,ecn,eue,eric,8gh,lgs,mdc,kah,mlf,pif,bwh,ram,30h,nsm&expanders=concept&limiters=FT:Y&q=digital%20intervention%20OR%20mobile-based%20intervention%20OR%20mobile%20application%20OR%20smartphone%20app%20OR%20eHealth%20OR%20mHealth%20OR%20wearable%20device%20OR%20virtual%20intervention&searchMode=boolean&sort=relevance&sqId=sq:fe1146e7-27bc-4a69-9c5f-66c309772275&userDirectAction=true) 162,318

S4:[S1 AND S2 AND S](https://research.ebsco.com/search/results?combinedSearchQueryId=sq:1da10a6a-841b-40fa-a9a0-d2fd4b650a06&db=bth,ccm,cul,ddh,ecn,eue,eric,8gh,lgs,mdc,kah,mlf,pif,bwh,ram,30h,nsm&expanders=concept&limiters=FT:Y&q=S4%20AND%20S3%20AND%20S2&searchMode=boolean&sort=relevance&sqId=sq:1da10a6a-841b-40fa-a9a0-d2fd4b650a06&userDirectAction=true)3 102
